# Identifying Chemical Reactions and Their Associated Attributes in Patents

**DOI:** 10.3389/frma.2021.688353

**Published:** 2021-07-12

**Authors:** Darshini Mahendran, Gabrielle Gurdin, Nastassja Lewinski, Christina Tang, Bridget T. McInnes

**Affiliations:** ^1^ Department of Computer Science, Virginia Commonwealth University, Richmond, VA, United States; ^2^ Department of Life Science and Chemical Engineering, Virginia Commonwealth University, Richmond, VA, United States

**Keywords:** named entity recognition, event extraction, relation extraction, information extraction, chemical natural language processing

## Abstract

Chemical patents are an essential source of information about novel chemicals and chemical reactions. However, with the increasing volume of such patents, mining information about these chemicals and chemical reactions has become a time-intensive and laborious endeavor. In this study, we present a system to extract chemical reaction events from patents automatically. Our approach consists of two steps: 1) named entity recognition (NER)—the automatic identification of chemical reaction parameters from the corresponding text, and 2) event extraction (EE)—the automatic classifying and linking of entities based on their relationships to each other. For our NER system, we evaluate bidirectional long short-term memory (BiLSTM)-based and bidirectional encoder representations from transformer (BERT)-based methods. For our EE system, we evaluate BERT-based, convolutional neural network (CNN)-based, and rule-based methods. We evaluate our NER and EE components independently and as an end-to-end system, reporting the precision, recall, and *F*_1_ score. Our results show that the BiLSTM-based method performed best at identifying the entities, and the CNN-based method performed best at extracting events.

## 1 Introduction

Chemical patents are a significant source of information about novel chemicals and chemical reactions. New chemical compound discovery plays a vital role in the chemical and pharmaceutical industry, and chemical patents are the first venue this information is disclosed (He et al., 2021). Unfortunately, there has been a rapid growth of chemical patents in recent years, and with the increasing volume, the manual cataloging of these chemicals and chemical reactions is become laborious and time-intensive, making it difficult for researchers to keep up with the current state of the art. This has created an urgent need for automated solutions to extract information from patents in order to expedite the work of synthetic chemists (Lowe and Mayfield, 2020). Furthermore, these databases allow for the discovery of new chemical and synthetic pathways (Wang et al., 2001; Bort et al., 2020).

A chemical reaction typically includes an ordered sequence of reaction and workup steps that transforms a starting material into an end product (He et al., 2021). The process of extracting these steps consists of two key tasks: chemical named entity recognition (NER) and event extraction (EE). NER is the automatic identification of entities involved in a chemical reaction, and EE is the automatic identification and classification of event steps that link entities together. Here, the entities are names of chemical compounds labeled based on their role in a reaction and conditions associated with the reaction, such as yield and temperature (Copara et al., 2020), and the event is the relation between the entities that describe the steps taken to create the final product. To identify the entities and trigger words, we explored bidirectional long short-term memory (BiLSTM)-based and bidirectional encoder representations from transformer (BERT)-based (Devlin et al., 2018) methods each combined with a conditional random field (CRF) output layer for the final prediction. To identify the events, we explored rule-based, convolutional neural network (CNN)-based, and BERT-based methods. We evaluated our methods on the CLEF ChEMU-2020 dataset (He et al., 2021) where we also participated in the challenge (Mahendran et al., 2020). We evaluated each of the methods on their individual tasks (NER and EE) independently, and as an end-to-end system. We reported the precision, recall, and *F*_1_ score finding our best method (BiLSTM + CRF) for NER obtained an overall relaxed precision of 0.95 and exact precision of 0.87, relaxed recall of 0.99 and exact recall of 0.85, and a relaxed *F*_1_ score of 0.97 and an exact *F*_1_ score of 0.87. Our best method for EE (CNN-based) obtained an overall precision of 0.81, recall of 0.54, and *F*_1_ score of 0.65.

The remainder of the study is as follows. First, we discuss the current literature on extracting chemical reactions for patents. Second, we describe our NER and EE methods. Third, we discuss and analyze the results of our NER and EE separately, and then the results of combining them into an end-to-end system. Finally, we discuss the conclusions and future work of our research.

## 2 Related Work

The extraction of chemical reaction properties and events from unstructured text is essential due to the increasing volume of information. We define properties as the entities associated with the reaction, and the events as sequence of steps that transform a starting material into an end product. Here, we discuss the previous literature within this domain.

He et al. (2021) used a CRF-based model for NER and a rule-based system for EE. For NER, they developed the BANNER NER system (Leaman and Gonzalez, 2008) which uses lexical, syntactic, and contextual features in a CRF model. For EE, they used a co-occurrence–based method where they created two dictionaries: $D_{e}$—for the observed trigger words and their corresponding types, and $D_{r}$—for the observed event types from the training and development sets. To predict events, they first identified all trigger words in the test data using $D_{e}$ and then extracted two events for a trigger word–entity pair based on the following two conditions: 1) they co-occur in the same sentence, and 2) the relation is included in $D_{r}$. This system was utilized as the baseline system for the CLEF ChEMU-2020 challenge.

Copara et al. (2020) and Malarkodi et al. (2020) each developed a NER system to identify chemical entities from patents. Copara et al. (2020) used a BERT-based method assessing five variations of the BERT language models, including a domain-specific model called ChemBERTa. The models have a fully connected layer on top of the hidden states of each token and fine-tuned on the ChEMU dataset, using the training and development sets provided. Malarkodi et al. (2020) investigated using CRFs and multilayer perceptrons (MLPs), and they used word-level features, grammatical features, and functional term features.

Lowe and Mayfield (2020), Zhang and Zhang (2020), Ruas et al. (2020), and Dönmez et al. (2020) developed both NER and EE systems to extract chemical entities, and their trigger words, and subsequently link the trigger words to the entities to identify events. However, only Lowe and Mayfield (2020) and Zhang and Zhang (2020) conducted an end-to-end evaluation.

Lowe and Mayfield (2020) proposed a method utilizing parsing information with grammar rules for both NER and EE. For NER, they used ChemicalTagger (Hawizy et al., 2011) for efficient matching against extensive grammars that describe entity types to recognize chemicals and physical quantities, and then used regular expressions to recognize the remainder of the entity types and trigger words. For EE, they associated all of the entities within a phrase to its corresponding trigger word, with some predefined exception rules.

Zhang and Zhang (2020) proposed a hybrid combination of deep learning models and pattern-based rules for both NER and EE. In their work, a new language model, named Patent_BioBERT, was generated by pretraining the patent texts over BioBERT (Lee et al., 2019). For NER, they fine-tuned Patent_BioBERT on a BiLSTM + CRF and post-processed the output utilizing a set of pattern rules. For EE, they built a binary classifier by fine-tuning Patent_BioBERT to recognize relations between the trigger words and entities. They also designed post-processing rules based on patterns observed in the training data and applied them to recover some of these false-negative relations.

Ruas et al. (2020) proposed a BERT-based method for NER and EE. For NER, they first used a rule-based tokenizer for text tokenization and then a BERT-based model to extract the entities evaluating both BioBERT and BERT. For EE, they first performed sentence segmentation masking the trigger words, and the segment is then fed into a BERT model to classify the relation. Dönmez et al. (2020) proposed a BERT-based method for NER and a rule-based method for EE. For NER, they used the pretrained BERT model, BioBERT, to detect the entities and trigger words. For RE, if there is a trigger word in the same sentence as an entity, the event is identified based on a set of rules.

In this work, we analyze and benchmark different approaches for both NER and EE. For NER, we explore a BiLSTM + CRF and a BioBERT-based method. For EE, we explore a rule-based method that utilizes the colocation information, a CNN-based method that divides a sentence into segments and processes each segment unit separately, and two BERT-based methods. We evaluate the methods for NER and EE individually as well as an end-to-end system, conducting a thorough analysis of our results to identify the areas in which each approach does well and falls short.

## 3 Data

**FIGURE 1:**
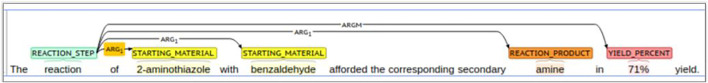
An example from the CLEF ChEMU-2020 dataset that shows the entities, trigger words, and events.

The CLEF ChEMU-2020 dataset contains patents annotated with chemical entities and events explaining the sequence of steps that lead a starting material through a chemical reaction to an end product (Nguyen et al., 2020). It includes ten entities and two event classes. Table 1 displays the definitions of each entity and trigger word label in detail. Table 2 shows the event statistics of the training dataset. The dataset includes two trigger words (REACTION_STEP and WORKUP), and an event consists of a trigger word and an entity. Events are divided into two classes: ARG1 and ARGM. The ARG1 event label corresponds to relations between a trigger word and chemical compound entities. The ARGM event label corresponds to the relations between a trigger word and temperature, time, or yield entities. Figure 1 shows an example of a sentence from the CLEF ChEMU-2020 dataset (He et al., 2021) that explains the relationship between an entity and a trigger word.

**TABLE 1:** Definitions of entities and trigger words of the dataset.

Entity labels	Definition
REACTION_PRODUCT (R.P.)	A product is a substance that is formed during a chemical reaction
STARTING_MATERIAL (S.M.)	A substance that is consumed in the course of a chemical reaction providing atoms to products is considered as starting material
REAGENT_CATALYST (R.C.)	A reagent is a compound added to a system to cause or help with a chemical reaction. Compounds like catalysts, bases to remove protons, or acids to add protons must also be annotated with this tag
SOLVENT (S)	A solvent is a chemical entity that dissolves a solute, resulting in a solution
OTHER_COMPOUND (O.C.)	Other chemical compounds that are not the products, starting materials, reagents, catalysts, and solvents
TIME	The reaction time of the reaction
TEMPERATURE (Temp)	The temperature of the reaction
YIELD_PERCENT (Y.P.)	Yields given in percent values
YIELD_OTHER (Y.O.)	Yields provided in other units than %
EXAMPLE_LABEL	A label associated with a reaction specification
REACTION_STEP	Event within which starting materials are converted into product
WORKUP	Event step required to isolate and purify the product of a chemical reaction

**TABLE 2:** Number of entity types and trigger words in the training data and their event relations.

Events	Entities	Instances	REACTION_STEP	WORKUP
ARG1	EXAMPLE_LABEL	886	—	—
	REACTION_PRODUCT	2052	1,101	11
	STARTING_MATERIAL	1754	1747	4
	REAGENT_CATALYST	1,281	1,272	—
	SOLVENT	1,140	1,134	4
	OTHER_COMPOUND	4,640	161	4,097
ARGM	YIELD_PERCENT	955	937	1
	YIELD_OTHER	1,061	1,043	2
	TIME	1,059	839	81
	TEMPERATURE	1,515	813	242
Triggers	REACTION_STEP	3,815		
	WORKUP	3,053		

## 4 Methods

This section describes the underlying methodology of our NER and EE systems.

### 4.1 Named Entity Recognition and Trigger Detection

We evaluate using BiLSTMs and a BERT to identify the chemical entities and their trigger words. In this section, we describe each of these in detail.

#### 4.1.1 Bidirectional Long Short Term Memory

For our BiLSTM method, we use BiLSTM units with a CRF output layer (BiLSTM + CRF). An LSTM (Huang et al., 2015) is a type of recurrent neural network (RNN). It has two sources of input: the current state and the past states. This allows the cell to connect the previous observations, such as words in a sentence, and learn the dependencies of these words over arbitrarily long distances. The LSTM identifies what information should be passed to the next component, allowing only the relevant information. With BiLSTMs, data are processed in both directions with two separate hidden layers to enable the system to exploit context in both directions. We use a linear-chain CRF to assign the final class probability. CRF is a sequence-learning algorithm that incorporates the interdependence between labels into model induction and prediction. This allows the model to use the preceding label predictions to inform what labels are most likely to follow or to occur close together.

We represent our input into the BiLSTM + CRF using pretrained word embeddings (Mikolov et al., 2013) in combination with character embeddings (Gridach, 2017). We use the pretrained ChemPatent embeddings (Nguyen et al., 2020) that are trained over a collection of 84,076 full patent documents (1B tokens). The word and character embeddings are then concatenated and then passed through the network. The character embeddings are learned using a BiLSTM layer and concatenated onto the word embeddings. The character embeddings are valuable for alleviating the problem of “out of vocabulary” (OOV) terms for the model. In the case of chemical patents, many tokens are long chemical names that do not show up in the dataset used to train word embeddings, such as the reaction product *3-Isobutyl-5-methyl-1-(oxetane-2-ylmethyl)-6-[(2-oxoimidazolidin-1-yl)methyl]thieno[2,3-d]pyrimidine-2,4(1H,3H)-dione*.

#### 4.1.2 Bidirectional Encoder Representations From Transformers

For our BERT method, we use BERT’s embedding representation into a feed-forward neural network with a CRF output layer. BERT is a contextualized word embedding model trained over a large corpus for masked language modeling and next sentence prediction tasks. Devlin et al. (2018) showed that this pretrained model could then be fine-tuned for other NLP tasks, including NER, by adding a simple classification layer. We evaluated BERT and several other additions for extracting chemical reaction parameters from chemical patents. Our architecture consists of an 1) alternate WordPiece labeling component, 2) BioBERT feature representation, and 3) a simple feed-forward layer into a CRF output layer for the final prediction.

Alternate WordPiece labeling: BERT tokenization involves splitting some tokens into “WordPieces” referred to as subwords, which alleviates the problem of OOV words. However, this creates a complication when doing token-level classification like NER; as per Devlin et al. (2018) recommendation, we only classify the first WordPiece by masking the rest and applying an “X” label.

BioBERT: BioBERT (Lee et al., 2019) is a BERT model that was further pretrained over biomedical text. In our work, the input to BioBERT is a single text sentence that is broken into subwords. An input representation is constructed by integrating the token, segment, and positional embeddings. Lee et al. (2019) started by loading the BERT-based cased weights and then training over PubMed abstracts and PubMed Central articles. For our experiments, we loaded the BioBERT v1.1 weights and then fine-tuned the model identically to standard BERT.

Classification layers: The system takes the output of the BioBERT layers, and passes it through a simple dense feed-forward layer and then into the CRF layer for the final classification. The CRF layer allows for the dependencies between the labels to be incorporated into the final prediction.

### 4.2 Event Extraction

Since a chemical reaction step involves action and chemical compound(s) on which the action takes effect, we treat EE as a two-stage task: 1) identification of a trigger word that indicates a chemical reaction step and 2) identification of the relation between a trigger word and chemical compound(s) that is (are) linked to the trigger word.

To identify the trigger words, we use our NER system BiLSTM + CRF method described in Section 4.1.1. To identify the relations between the trigger words and the entities, we explore three methods: 1) rule-based method, 2) convolutional neural network(CNN)-based method, and 3) BERT-based methods: BERT_cased and BioBERT (Lee et al., 2019). In this section, we describe each of the methods in detail.

#### 4.2.1 Rule-Based Method

For our rule-based method, we utilize the colocation information between the trigger word and the chemical entity to determine if a relation should exist. We use a breadth-first search algorithm to find the trigger word’s closest occurrence on either side of the entity and all the closest occurrences of the trigger words within a sentence. Then, for each entity in the dataset, we traverse both sides until the trigger word’s most immediate occurrence is found using the provided span values of the entities. We apply different traversal techniques and determine the best traversal technique. The following are the traversal techniques we explore: traverse left-only, traverse right-only, traverse left-first-then-right, and *vice versa*. In this work, we report the best results, which used the left-only traversal where we traverse to the left side of the entity mention finding the closest occurrence of the trigger words.

#### 4.2.2 CNN-Based Method

For our CNN-based method, we split the sentence into segments and pass each segment into its respective CNN architecture, joining the resulting weights into a softmax layer for classification. CNNs are a form of deep neural networks and consist of four main layers (Nguyen and Grishman, 2015): 1) an embedding layer, 2) a convolution layer, 3) a pooling layer, and 4) a feed-forward layer. The convolution layer acts as a filter and learns what features to extract from the input. The max-pooling layer uses the position information to identify the most significant features from the convolution filter’s output. Finally, the feed-forward layer performs classification.

In our architecture, we perform a binary classification for each trigger word–entity pair to identify whether a relation exists between the trigger word and the entity. First, we identify and extract the sentence where a trigger word–entity pair lies, and based on where the text spans are located in the sentence, we divide the sentence into five segments: 1) preceding—tokenized words before the first concept, 2) concept 1—tokenized words in the first concept, 3) middle—tokenized words between the two concepts, 4) concept 2—tokenized words in the second concept, and 5) succeeding—tokenized words after the second concept. A segment is represented by a matrix of k∗N, where *k* is the dimension of the word embeddings and *N* is the number of words in a segment. We construct separate convolution units for each segment and concatenate them before the fixed-length vector is fed to the dense layer that performs the classification. Each convolution unit applies a sliding window that processes the segment and feeds the output to the max-pooling layer to extract essential features independent of their location. The output features of the max-pooling layer of each segment are then flattened and concatenated into a vector before feeding it into the fully connected feed-forward layer. The vector is finally fed into a softmax layer to perform the binary classification on whether the relationship exists.

#### 4.2.3 BERT-Based Method

For our BERT-based methods, we explore two BERT-contextualized embedding representations. BERT is a transformer-based attention model which trains in both directions. Here, we use BERT-contextualized embeddings and feed them into a simple feed-forward neural network. We explore two BERT-based models: BERT_cased and BioBERT.

• BERT_cased: general BERT models trained on a large corpus of English data: Book-Corpus (800 M words) and Wikipedia (2,500 M words) in a self-supervised manner (without human annotation). Here, we used the model with 2 heads, 12 layers, 768 hidden units/layers, and a total of 110 M parameters.
• BioBERT: general BERT model, further trained over a corpus of biomedical research articles from PubMed^1^ abstracts and PubMed Central^2^ article full texts.

In this architecture, we first extract the sentences that contain a trigger word and entity arguments. Next, as our feature extraction component, we pass the sentences through the pretrained BERT models to extract the features. Then, we feed the output into a dropout layer and finally into a fully connected dense layer for classification. As with our CNN-based method, we treat the EE as a binary classification task building a separate model for each trigger word–entity pair.

### 4.3 Experimental Details

#### 4.3.1 Our Framework

In this work, we used our NER and EE frameworks: MedaCy and RelEx.

MedaCy^3^ is a python-based framework developed to automatically identify the experimental parameters associated with the reaction, including the trigger words. RelEx^4^ is a python-based framework developed to automatically link the trigger words with the experimental parameters to provide the sequence of steps within the reaction. MedaCy contains a number of supervised multi-label sequence classification algorithms for NER. RelEx contains rule-based, deep learning–based, and BERT-based algorithms to identify relations between entities.

MedaCy: we used PyTorch (Paszke et al., 2019) for the implementation of the BiLSTM + CRF and BioBERT + CRF architectures. The models were trained for 40 epochs and optimized using stochastic gradient descent. Tokenization was conducted using the SpaCy tokenizer. The labels are strictly the entity types.

RelEx: we used Keras (Charles, 2013) for the implementation of the CNN architecture. We experimented with different sliding window sizes, filter sizes, and loss functions for fine-tuning, and in this work, small filter sizes generated the best results. We applied the dropout technique on the output of the convolution layer to regularize the model. We used Adam and rmsprop optimizers to minimize our loss function. We utilized SpaCy tokenizer (Schatz and Weber, 2015) and Keras tokenizer^5^ for the rule-based and the CNN-based method, respectively. We trained the models for 5–10 epochs to avoid over-fitting. We used the HuggingFaceTransformers to build the BERT models from Tensorflow 2.0, and used BertTokenizer (Devlin et al., 2018) and AutoTokenizer (Alsentzer et al., 2019) for tokenization.

### 4.4 Evaluation

We report the precision, recall, and *F*_1_ scores. Precision is the ratio between correctly predicted mentions over the total set of predicted mentions for a specific entity, recall is the ratio of correctly predicted mentions over the actual number of mentions, and *F*_1_ is the harmonic mean between precision and recall. We also report both the exact and relaxed results for each entity category for our NER and end-to-end evaluation. In the exact evaluation, two annotations are equal only if they have the same tag with exactly matching spans. With the relaxed evaluation, two annotations are equal if they share the same tag and their spans overlap.

## 5 Results and Discussion

In this section, we present and discuss the results of our NER and EE systems evaluated independently and then as a complete end-to-end system.

### 5.1 Named Entity Recognition Results

Table 3 shows the exact and relaxed precision (P), recall (R), and *F*_*1*_ (F) scores obtained over the test set for our BiLSTM + CRF with the ChEMU patent embeddings and our BioBERT + CRF methods.

**TABLE 3:** Precision (P), recall (R), and *F*_1_ (F) results for our NER system.

Method	Entity	Exact			Relax		
		P	R	F	P	R	F
BiLSTM + CRF	EXAMPLE_LABEL	0.94	0.95	0.94	0.94	0.98	0.96
	OTHER_COMPOUND	0.9	0.82	0.86	0.97	0.99	0.98
	REACTION_PRODUCT	0.84	0.83	0.83	0.9	0.97	0.94
	REAGENT_CATALYST	0.85	0.9	0.87	0.88	0.99	0.93
	SOLVENT	0.91	0.94	0.93	0.92	1	0.96
	STARTING_MATERIAL	0.85	0.84	0.85	0.91	1	0.95
	TEMPERATURE	0.63	0.63	0.63	0.99	0.99	0.99
	TIME	0.88	0.88	0.88	1	1	1
	YIELD_OTHER	0.95	0.98	0.97	0.96	1	0.98
	YIELD_PERCENT	0.99	0.99	0.99	1	1	1
		System	**0.87**	**0.85**	**0.86**	**0.95**	**0.99**	**0.97**
BioBERT + CRF	EXAMPLE_LABEL	0.91	0.94	0.92	0.92	0.95	0.94
	OTHER_COMPOUND	0.88	0.83	0.85	0.95	0.94	0.95
	REACTION_PRODUCT	0.44	0.65	0.52	0.73	0.95	0.82
	REAGENT_CATALYST	0.78	0.81	0.79	0.86	0.87	0.87
	SOLVENT	0.89	0.92	0.90	0.90	0.92	0.91
	STARTING_MATERIAL	0.39	0.60	0.48	0.69	0.92	0.79
	TEMPERATURE	0.95	0.96	0.96	0.98	0.99	0.99
	TIME	0.88	0.88	0.88	0.99	0.99	0.99
	YIELD_OTHER	0.78	0.85	0.81	0.89	0.95	0.92
	YIELD_PERCENT	0.95	0.99	0.97	0.97	1.00	0.98
		System	**0.73**	**0.82**	**0.77**	**0.87**	**0.95**	**0.91**

Bold indicates system performance of both models.

In the BiLSTM + CRF results, the exact F-1 score was high (≥0.85) across all entities, except for the exact results for TEMPERATURE (0.63). In many cases of the TEMPERATURE label, the model labeled “C” or “°C,” excluding the number preceding the temperature symbol. We believe this accounts for the model performed poorly when evaluating in the exact mode but performed well when evaluating in the relaxed mode.

In the BioBERT + CRF results, the model performed on par with the BiLSTM + CRF except for REACTION_PRODUCT and STARTING_MATERIAL. The relaxed results indicate that the model is labeling a portion of the entity and can identify most of them; however, the precision is relatively low. BioBERT initially obtains a label for each subword token. These are then combined to provide both token-level predictions, which are fed into the CRF layer to obtain the final entity-level predictions. This allows for dependencies between the entities to be taken into consideration for sequence labeling. The BioBERT results indicate that it is getting most of an entity but not all of it; this suggests that post-processing of the labels can be improved in the future to obtain a full exact match.

#### 5.1.1 Error Analysis

**FIGURE 2:**
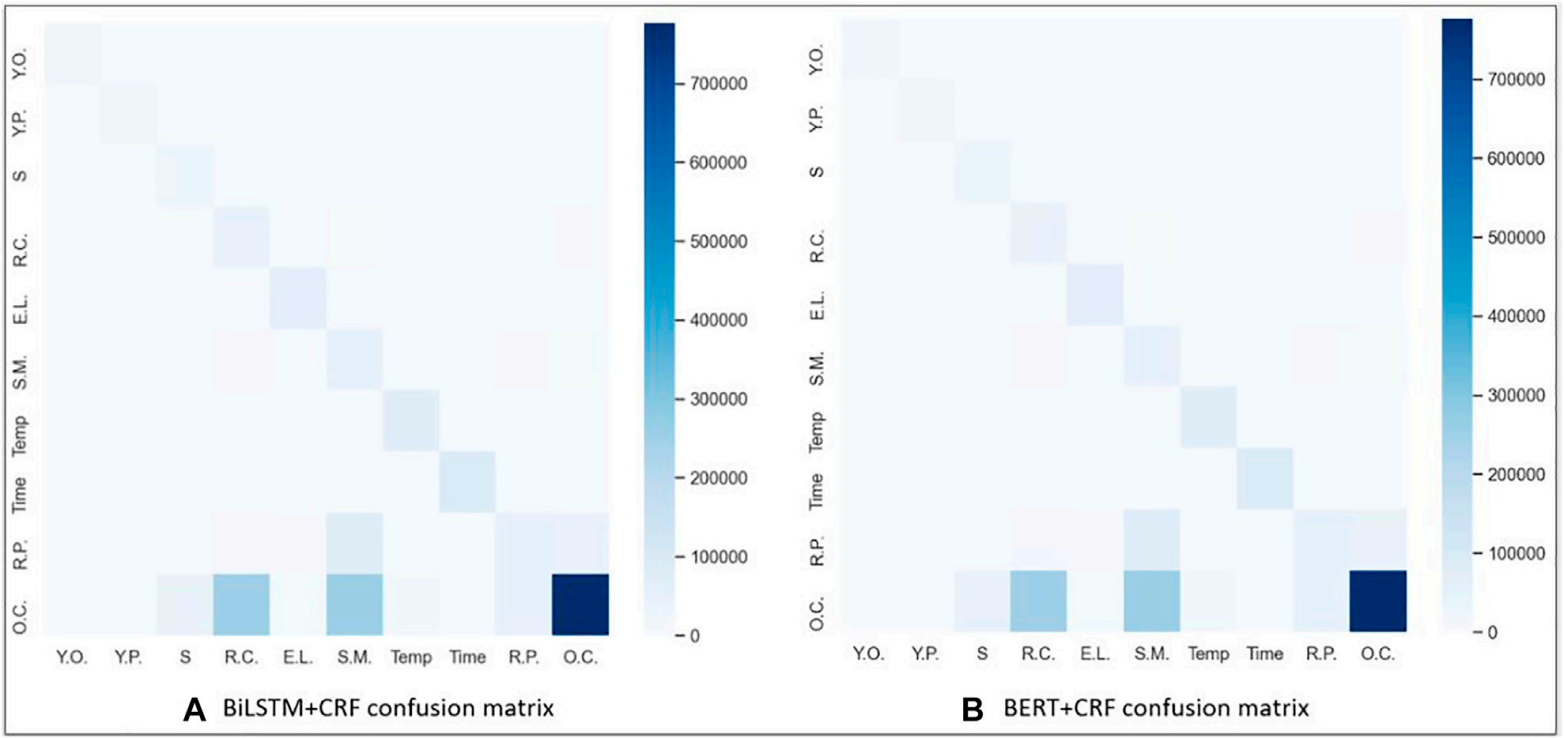
Confusion matrix using **(A)** BiLSTM + CRF and **(B)** BERT + CRF results. Keys for the acronyms are as follows: EXAMPLE_LABEL (E.L.), REACTION_PRODUCT (R.P.), STARTING_MATERIAL (S.M.), REAGENT_CATALYST (R.C.), SOLVENT (S), OTHER_COMPOUND (O.C.), YIELD_PERCENT (Y.P.), YIELD_OTHER (Y.O.), TIME (Time), and TEMPERATURE (Temp).

Confusion matrices for the BiLSTM + CRF and BERT + CRF over the testing dataset are shown in Figure 2. Rows in the matrix represent annotated entities, and columns represent predicted entities. For instance, in the BiLSTM + CRF, the bottom right corner of each matrix is darker because of the large number of OTHER_COMPOUND (O.C) entities in the dataset. The colors in the matrix indicate the density of the entities and the system annotations. The matrix shows that the majority of mislabeling for both models occurred when many of the specific entity labels, such as STARTING_MATERIAL (S.M.), REAGENT_CATALYST (R.C.), REACTION_PRODUCT (R.P.), and SOLVENT (S), were predicted to be OTHER_COMPOUND (O.C.), as all four of those entities are chemicals. We believe this is due to two main reasons. The first reason is obvious; there is a significantly larger number of training instances for OTHER_COMPOUND than the other entities. However, the second reason is that OTHER_COMPOUND is quite a broad category referring to any chemical that is not one of the other four entities. Therefore, if the context surrounding the chemical is not sufficient to place it in the STARTING_MATERIAL, REAGENT_CATALYST, REACTION_PRODUCT, or SOLVENT label, it defaults to the broader OTHER_COMPOUND label.

#### 5.1.2 Comparison With a Previous Work

Table 4 shows a comparison between the top results reported by the CLEF ChEMU-2020 challenge using the CLEF-2020 dataset, baseline, and our NER methods. Baseline is a CRF-based NER system called BANNER (Leaman and Gonzalez, 2008) provided by the ChEMU organizers using the CLEF-2020 dataset. From the overall results of our models, we can see the BiLSTM + CRF method trained using patent embeddings returned the best relaxed results over both the BioBERT + CRF and the CRF baseline, obtaining a 97% system-wide relaxed + score, however, scoring slightly lower on the exact results than on the baseline. Melaxtech (Zhang and Zhang, 2020) fine-tuned the BioBERT over the patent texts and a BiLSTM + CRF for NER; they outperformed other systems, achieving a high F1-score of 0.96. VinAI (Dao and Nguyen, 2020), Lasige BioTM (Ruas et al., 2020), and BiTeM (Copara et al., 2020) performed equally and better than our methods under exact match. Our BiLSTM + CRF method outperformed the baseline and other methods under relaxed match, achieving higher recall. As discussed in the related work (Section 2), MelaxTech, BiTeM, and LasigBioTM developed BERT-based systems; MelaxTech also used a BiLSTM + CRF as VinAI. AU-KBC built systems using CRFs and MLP.

**TABLE 4:** Our best results in comparison with the top results of the ChEMU-2020 competition for NER. Baseline is provided by the organizers of the ChEMU-2020 challenge.

		Exact			Relax		
		P	R	F	P	R	F
Our methods	BiLSTM-based	0.87	0.85	0.86	0.95	**0.99**	**0.97**
	BioBERT-based	0.73	0.82	0.77	0.87	0.95	0.91
ChEMU_2020 teams	Melaxtech Zhang and Zhang (2020)	**0.96**	**0.96**	**0.96**	**0.97**	0.97	**0.97**
	VinAI Dao and Nguyen (2020)	0.95	0.95	0.95	**0.97**	0.97	**0.97**
	Lasige BioTM Ruas et al. (2020)	0.93	0.95	0.94	0.96	0.97	0.96
	BiTeM Copara et al. (2020)	0.94	0.91	0.92	**0.97**	0.96	0.96
	NextMove/Minesoft Lowe and Mayfield (2020)	0.90	0.89	0.90	0.93	0.92	0.92
	AUKBC Malarkodi et al. (2020)	0.68	0.41	0.51	0.88	0.53	0.66
Baseline	ChEMU organizers	0.91	0.87	0.89	0.92	0.89	0.91

Bold indicates best results for P, R, and F for both exact and relax match results.

### 5.2 Event Extraction Results

Table 5 shows the exact match precision (P), recall (R), and *F*_*1*_ (F) scores obtained for the EE system. The triggers were identified using our BiLSTM + CRF method trained over the ChemPatent embeddings, and the events were identified using our rule-based method, our CNN-based method, and our two BERT-based methods. The results show that the CNN-based method obtained a higher overall *F*_1_ score than the other methods. When training with CNN, the overall precision of the predictions is high, but the recall is low; this result shows that CNN failed to classify all instances but was able to classify most of the predicted instances correctly. This is primarily due to the limited number of training instances for many of the WORKUP relations. For example, SOLVENT, REACTION_PRODUCT, and STARTING_MATERIAL all have less than 11 instances in the training data.

**TABLE 5:** Precision (P), recall (R), and $F_{1}$ (F) score of the EE system with trigger words identified using our BiLSTM + CRF trained with ChEMU patent embeddings.

Method	Argument	Trigger	Entity	# Train	P	R	F
Rule-based	ARG1	REACTION_STEP	OTHER_COMPOUND	161	0.02	0.06	0.04
			REACTION_PRODUCT	1,101	0.82	0.78	0.80
			REAGENT_CATALYST	1,272	0.52	0.35	0.42
			SOLVENT	1,134	0.81	0.55	0.65
			STARTING_MATERIAL	1747	0.63	0.31	0.41
			Average		0.56	0.52	0.46
		WORKUP	OTHER_COMPOUND	4,097	0.90	0.86	0.88
			REACTION_PRODUCT	11	0.01	1.00	0.02
			REAGENT_CATALYST	—	0.00	0.00	0.00
			SOLVENT	4	0.07	1.00	0.14
			STARTING_MATERIAL	4	0.04	1.00	0.08
			Average		0.20	0.77	0.22
	ARGM	REACTION_STEP	TEMPERATURE	813	0.77	0.89	0.83
			TIME	839	0.85	0.93	0.89
			YIELD_OTHER	1,043	0.83	0.80	0.81
			YIELD_PERCENT	937	0.86	0.85	0.85
			Average		0.83	0.87	0.85
		WORKUP	TEMPERATURE	242	0.66	0.81	0.73
			TIME	81	0.36	0.53	0.43
			Average		0.51	0.67	0.58
	System				**0.51**	**0.72**	**0.60**
CNN-based	ARG1	REACTION_STEP	OTHER_COMPOUND	161	0.00	0.00	0.00
			REACTION_PRODUCT	1,101	0.92	0.96	0.94
			REAGENT_CATALYST	1,272	0.78	0.69	0.74
			SOLVENT	1,134	0.64	0.74	0.69
			STARTING_MATERIAL	1747	0.82	0.43	0.56
			Average	—	0.63	0.56	0.59
		WORKUP	OTHER_COMPOUND	4,097	0.73	0.29	0.42
			REACTION_PRODUCT	11	0.00	0.00	0.00
			SOLVENT	4	0.00	0.00	0.00
			STARTING_MATERIAL	4	0.00	0.00	0.00
			Average		0.18	0.07	0.11
	ARGM	REACTION_STEP	TEMPERATURE	813	0.83	0.30	0.44
			TIME	839	0.78	0.73	0.75
			YIELD_OTHER	1,043	0.93	0.96	0.95
			YIELD_PERCENT	937	0.91	0.94	0.92
			Average		0.86	0.73	0.77
		WORKUP	TEMPERATURE	242	0.56	0.08	0.14
			TIME	81	0 .00	0.00	0.00
			Average		0.28	0.04	0.07
	System				**0.81**	**0.54**	**0.65**
BERT-based	ARG1	REACTION_STEP	OTHER_COMPOUND	161	0.03	0.06	0.04
			REACTION_PRODUCT	1,101	0.84	0.82	0.83
			REAGENT_CATALYST	1,272	0.51	0.2	0.29
			SOLVENT	1,134	0.49	0.62	0.55
			STARTING_MATERIAL	1747	0.55	0.92	0.69
			Average		0.48	0.52	0.59
		WORKUP	OTHER_COMPOUND	4,097	0.54	0.48	0.51
			REACTION_PRODUCT	11	0.00	0.00	0.00
			SOLVENT	4	0.00	0.00	0.00
			STARTING_MATERIAL	4	0.00	0.00	0.00
			Average		0.14	0.12	0.13
	ARGM	REACTION_STEP	TEMPERATURE	813	0.44	0.20	0.27
			TIME	839	0.51	0.82	0.63
			YIELD_OTHER	1,043	0.83	0.83	0.83
			YIELD_PERCENT	937	0.84	0.92	0.88
			Average		0.66	0.69	0.65
		WORKUP	TEMPERATURE	242	0.26	0.17	0.21
			TIME	81	0.23	0.26	0.24
			Average		0.25	0.22	0.23
	System				**0.58**	**0.59**	**0.58**
BioBERT-based	ARG1	REACTION_STEP	OTHER_COMPOUND	161	0.04	0.02	0.02
			REACTION_PRODUCT	1,101	0.84	0.82	0.83
			REAGENT_CATALYST	1,272	0.53	0.45	0.49
			SOLVENT	1,134	0.51	0.39	0.44
			STARTING_MATERIAL	1747	0.59	0.27	0.37
			Average		0.50	0.39	0.43
		WORKUP	OTHER_COMPOUND	4,097	0.52	0.53	0.54
			REACTION_PRODUCT	11	0.00	0.00	0.00
			SOLVENT	4	0.00	0.00	0.00
			STARTING_MATERIAL	4	0.00	0.00	0.00
			Average		0.13	0.13	0.14
	ARGM	REACTION_STEP	TEMPERATURE	813	0.43	0.08	0.13
			TIME	839	0.57	0.30	0.40
			YIELD_OTHER	1,043	0.84	0.81	0.82
			YIELD_PERCENT	937	0.84	0.88	0.86
			Average	—	0.67	0.52	0.56
		WORKUP	TEMPERATURE	242	0.27	0.20	0.23
			TIME	81	0.17	0.02	0.04
			Average		0.22	0.11	0.14
	System				**0.62**	**0.50**	**0.55**

Bold indicates system performance of three methods.

We can also see that each event class performance (trigger word–entity pair) in the CNN-based method is proportional to the number of instances in the training set. For example, event classes REACTION_STEP—REAGENT_CATALYST, and REACTION_STEP–STARTING_MATERIAL have more training instances and obtain a high *F*_*1*_ score, whereas the event classes WORKUP-SOLVENT and WORKUP-STARTING_MATERIAL have a very few instances and obtain an *F*_1_ score of zero. The rule-based method obtains comparatively high recall but low precision. It predicts all the closest occurrences of the trigger words of the entity compounds in the traversal area; however, many predictions are false positives. Since the number of instances in the training set does not affect the rule-based methods, the event classes that have few instances perform better. For example, the event classes WORKUP-TIME and REACTION_STEP-OTHER_COMPOUND obtained zero *F*_1_ score with the CNN-based method but performed better with the rule-based method, obtaining *F*_1_ scores of 0.43 and 0.88, respectively.

Both BERT-based methods obtain mediocre results compared to other rule-based and CNN-based methods, which we find surprising. The precision of both BERT-based methods is higher than that of the rule-based method but lower than that of the CNN-based method, and the recall is lower than that of the rule-based method but higher than that of the CNN-based method. We assume the reasons behind these findings are the BERT-based methods utilize contextualized embeddings which improve the number of the predictions (high recall), but the CNN-based method utilizes domain-related, non-contextualized patent embeddings that improve the number of true positives (high precision). Also, BERT-based methods take only the sentence where the trigger word–entity pair is located as the input, whereas the CNN-based method breaks the sentence into segments and processes each segment separately. Therefore, the CNN-based method considers the positional information of the entities results in more true positives. If we compare the BERT-based methods with each other, we can see the BERT_cased method obtained a higher recall and an overall *F*_*1*_ score, whereas the BioBERT-based method obtained a high precision. Since the BioBERT embeddings are trained over biomedical research articles, comparatively, they classify most of the predicted instances correctly.

Each trigger word category shows the arithmetic mean for both trigger word classes for each entity argument class. We can see the CNN-based method performs well with the REACTION_STEP classes and poor with WORKUP classes. This is mainly because of the number of instances in each event class. Comparatively, most of the REACTION_STEP classes have more instances for the CNN to train than most WORKUP classes. This is the same reason the rule-based method performs better with the WORKUP classes. BERT-based method results are similar to those of the CNN-based method; they perform well with the REACTION_STEP classes compared to the WORKUP classes. Since both BERT-based and CNN-based methods are supervised learning methods, they need more instances for each class to improve the results.

#### 5.2.1 Error Analysis

Table 6 shows a detailed error analysis our EE methods. We report the number of true positives (tp), false positives (fp), and false negatives (fn), and also “fpm” and “fnm,” two metrics that represent the number of false positives and false negatives of the trigger words predicted.

**TABLE 6:** Error analysis for the event extraction (EE) system where trigger words are trained with ChemPatent embeddings.

Method	Argument	Trigger	Entity	tp	fp	fn	fpm	fnm
Rule-based	ARG1	REACTION_STEP	OTHER_COMPOUND	40	1798	23	18	11
			REACTION_PRODUCT	351	75	101	10	3
			REAGENT_CATALYST	177	162	328	8	8
			SOLVENT	234	54	193	4	7
			STARTING_MATERIAL	217	128	494	15	9
		WORKUP	OTHER_COMPOUND	1,501	171	249	54	73
			REACTION_PRODUCT	4	375	0	9	0
			REAGENT_CATALYST	0	40	0	9	0
			SOLVENT	2	25	0	5	0
			STARTING_MATERIAL	1	24	0	2	0
	ARGM	REACTION_STEP	TEMPERATURE	450	131	53	29	15
			TIME	386	66	27	21	10
			YIELD_OTHER	350	74	85	11	3
			YIELD_PERCENT	326	55	58	11	3
		WORKUP	TEMPERATURE	89	45	21	13	20
			TIME	23	41	20	16	13
System				4,151	3,957	1,652	421	175
CNN-based	ARG1	REACTION_STEP	OTHER_COMPOUND	0	0	63	0	11
			REACTION_PRODUCT	436	36	16	11	3
			REAGENT_CATALYST	350	97	155	17	8
			SOLVENT	316	179	111	16	7
			STARTING_MATERIAL	305	68	406	12	9
		WORKUP	OTHER_COMPOUND	516	192	1,234	23	73
			REACTION_PRODUCT	0	0	4	0	0
			REAGENT_CATALYST	—	—	—	—	—
			SOLVENT	0	0	2	0	0
			STARTING_MATERIAL	0	0	1	0	0
	ARGM	REACTION_STEP	TEMPERATURE	151	30	352	15	15
			TIME	300	87	113	16	10
			YIELD_OTHER	418	31	17	11	3
			YIELD_PERCENT	361	36	23	13	3
		WORKUP	TEMPERATURE	9	7	101	0	20
			TIME	0	0	43	0	13
System				3,162	763	2,641	134	175
BERT-based	ARG1	REACTION_STEP	OTHER_COMPOUND	4	120	59	15	11
			REACTION_PRODUCT	369	72	83	17	3
			REAGENT_CATALYST	101	97	404	9	8
			SOLVENT	266	273	161	22	7
			STARTING_MATERIAL	654	531	57	54	9
		WORKUP	OTHER_COMPOUND	845	708	905	77	73
			REACTION_PRODUCT	0	0	4	0	0
			REAGENT_CATALYST	—	—	—	—	—
			SOLVENT	0	0	2	0	0
			STARTING_MATERIAL	0	0	1	0	0
	ARGM	REACTION_STEP	TEMPERATURE	101	131	402	15	15
			TIME	338	319	75	18	3
			YIELD_OTHER	360	73	75	18	3
			YIELD_PERCENT	353	65	31	17	3
		WORKUP	TEMPERATURE	19	54	91	6	20
			TIME	11	37	32	0	13
System				3,421	2,480	2,382	284	175
BioBERT-based	ARG1	REACTION_STEP	OTHER_COMPOUND	0	10	63	2	11
			REACTION_PRODUCT	440	88	12	20	3
			REAGENT_CATALYST	156	146	349	13	8
			SOLVENT	256	235	171	20	7
			STARTING_MATERIAL	236	169	475	23	9
		WORKUP	OTHER_COMPOUND	928	790	822	73	68
			REACTION_PRODUCT	0	0	4	0	0
			REAGENT_CATALYST	—	—	—	—	—
			SOLVENT	0	0	2	0	0
			STARTING_MATERIAL	0	0	1	0	0
	ARGM	REACTION_STEP	TEMPERATURE	40	53	463	8	15
			TIME	95	95	288	7	10
			YIELD_OTHER	352	67	83	17	3
			YIELD_PERCENT	338	65	46	17	3
		WORKUP	TEMPERATURE	22	59	88	4	19
			TIME	1	5	42	0	13
System				2,984	1782	2,909	204	169

The results are consistent with the previous observations from Table 5. We can see that REACTION_STEP classes performed better than the WORKUP classes. It is safe to say that class imbalance plays a significant role in the miss-annotation of the instances. The results also show that the rule-based method significantly over annotates given the number of false positives. For example, the rule-based method identified 379 instances of the WORKUP-REACTION_PRODUCT event class, with only four being true positives. Despite having significant training instances in the REACTION_STEP classes, we can see an equally high number of false positives as true positives. This is mainly because extracting events is often trickier, regardless of the sentence pattern. For example, the following sentences show a trigger word–REACTION_PRODUCT pair in each.

1. After cooling, the solid was collected by filtration and washed with cold dichloromethane to give N-(4-(2-oxo-1,2,3,4-tetrahydroquinolin-6-yl)thiazol-2-yl)oxazole-5-carboxamide (0.121 g, 87%) as a beige solid.
2. {Methyl 4-[(6-bromo-2-phenyl-3-propylquinolin-4-yl)carbonyl]aminobicyclo[2.2.2]octane-1-carboxylate 150 mg (0.40 mmol) of the compound from example 38 A were dissolved in 1.4 ml (19.8 mmol) of thionyl chloride.

In the first sentence, the entity REACTION_PRODUCT N-(4-(2-oxo-1,2,3,4-tetrahydroquinolin-6-yl)thiazol-2-yl)oxazole-5-carboxamide is related to the trigger word *give*, but in the second sentence, the entity REACTION_PRODUCT {Methyl 4-[(6-bromo-2-phenyl-3-propylquinolin-4-yl)carbonyl]aminobicyclo[2.2.2]octane-1-carboxylate is not related to the trigger word dissolved. Despite the similar sentence structure, the results are not similar. These kinds of instances make the EE in this dataset quite hard.

In our EE methods, we utilized the trigger words predicted from our NER methods and the ground truth entities as the trigger words, respectively. From the metrics “fpm” and “fnm” for a trigger word–entity pair, we can see that when the number of “fpm” and “fnm” of a trigger word increases, the performance of the trigger word–entity pair decreases. We believe this is because the prediction of the trigger word–entity pair depends on the trigger word predicted by the biLSTM + CRF model described in Section 4.1.

#### 5.2.2 Comparison With a Previous Work

Table 7 shows a comparison between the top results reported by the CLEF ChEMU-2020 challenge using the CLEF-2020 dataset, the co-occurrence baseline provided by the organizers of the challenge, and the overall results of our EE methods. The overall results show that all three of our systems obtain a higher precision and *F*_1_ score than the baseline but not recall. Since the baseline method is a rule-based method based on the co-occurrence information, it obtains a high recall but low precision. Here, all systems outperform the baseline in terms of the *F*_1_ score, and Melaxtech (Zhang and Zhang, 2020) obtained the overall best performance using a hybrid combination of deep learning models and pattern-based rules for EE. As discussed in Section 2, NextMove/Minesoft Lowe and Mayfield (2020) proposed a method utilizing parsing information with grammar rules, and BOUN_REX (Dönmez et al., 2020) utilized a set of rules to identify the events. All teams performed better than our methods, except for the recall of Dönmez et al. (2020).

**TABLE 7:** Our best results in comparison with the top results of the ChEMU-2020 competition for event extraction (EE). Baseline is provided by the organizers of the ChEMU-2020 challenge.

		P	R	F
Our methods	Rule-based	0.51	0.72	0.60
	CNN-based	0.81	0.54	0.65
	BERT-based	0.58	0.59	0.58
	BioBERT-based	0.62	0.50	0.55
ChEMU_2020 teams	Melaxtech Zhang and Zhang (2020)	**0.96**	**0.95**	**0.95**
	NextMove/Minesoft Lowe and Mayfield (2020)	0.94	0.86	0.90
	BOUN_REX Dönmez et al. (2020)	0.76	0.69	0.72
Baseline	ChEMU organizers He et al. (2021)	0.24	0.89	0.38

Bold value results (P,R and F) of the best model from the competition.

### 5.3 End-to-End Results

An end-to-end system addresses both NER and EE; therefore, we combine our NER and EE methods to form a two-stage method. First, we use our BiLSTM + CRF method with the ChEMU patent embeddings, which produced the best results with NER to identify the entity arguments and trigger words. Then we use our CNN-based method with the ChEMU patent embeddings and the two BERT-based methods with the BERT contextualized embeddings to extract the events. From both the NER and EE results, we observed the precision of the deep-learning based methods is higher than that of the rule-based approach. Therefore, we decided to experiment only with deep-learning methods for the end-to-end system. Table 8 shows the exact and relax match precision (P), recall (R), and *F*_1_ (F) scores obtained for our three methods and shows a comparison between the top results reported by the ChEMU-2020 challenge participants using the CLEF-2020 dataset, the co-occurrence baseline provided by the organizers of the challenge, and the overall results of our end-to-end methods.

**TABLE 8:** Precision (P), recall (R), and $F_{1}$ (F) results for our end-to-end system using our BiLSTM + CRF NER and CNN-based EE methods.

Method	Argument	Trigger	Entity	Train	Exact			Relax		
					P	R	F	P	R	F
CNN-based	ARG1	REACTION_STEP	OTHER_COMPOUND	161	0.33	0.02	0.03	0.33	0.02	0.03
			REACTION_PRODUCT	1,101	0.72	0.79	0.76	0.81	0.92	0.86
			REAGENT_CATALYST	1,272	0.66	0.31	0.42	0.68	0.32	0.43
			SOLVENT	1,134	0.64	0.48	0.54	0.65	0.49	0.56
			STARTING_MATERIAL	1747	0.63	0.45	0.53	0.66	0.47	0.55
		WORKUP	OTHER_COMPOUND	4,097	0.67	0.61	0.64	0.72	0.69	0.70
			REACTION_PRODUCT	11	0.00	0.00	0.00	0.00	0.00	0.00
			SOLVENT	4	0.00	0.00	0.00	0.00	0.00	0.00
			STARTING_MATERIAL	4	0.00	0.00	0.00	0.00	0.00	0.00
	ARGM	REACTION_STEP	TEMPERATURE	813	0.43	0.15	0.22	0.62	0.21	0.31
			TIME	839	0.68	0.73	0.70	0.76	0.82	0.79
			YIELD_OTHER	1,043	0.81	0.92	0.86	0.86	0.96	0.91
			YIELD_PERCENT	937	0.85	0.95	0.90	0.86	0.96	0.91
		WORKUP	TEMPERATURE	242	0.25	0.12	0.16	0.41	0.19	0.26
			TIME	81	0.67	0.05	0.09	0.67	0.05	0.09
	System				**0.68**	**0.56**	**0.61**	**0.73**	**0.61**	**0.66**
BERT-based	ARG1	REACTION_STEP	OTHER_COMPOUND	161	0.08	0.05	0.06	0.08	0.05	0.06
			REACTION_PRODUCT	1,101	0.68	0.60	0.64	0.75	0.67	0.71
			REAGENT_CATALYST	1,272	0.45	0.48	0.46	0.48	0.5	0.49
			SOLVENT	1,134	0.45	0.18	0.25	0.46	0.18	0.26
			STARTING_MATERIAL	1747	0.48	0.77	0.59	0.51	0.84	0.63
		WORKUP	OTHER_COMPOUND	4,097	0.49	0.80	0.61	0.53	0.92	0.67
			REACTION_PRODUCT	11	0.00	0.00	0.00	0.00	0.00	0.00
			SOLVENT	4	0.00	0.00	0.00	0.00	0.00	0.00
			STARTING_MATERIAL	4	0.00	0.00	0.00	0.00	0.00	0.00
	ARGM	REACTION_STEP	TEMPERATURE	813	0.28	0.15	0.20	0.50	0.27	0.35
			TIME	839	0.43	0.38	0.40	0.49	0.44	0.46
			YIELD_OTHER	1,043	0.80	0.73	0.76	0.81	0.74	0.78
			YIELD_PERCENT	937	0.84	0.85	0.85	0.85	0.85	0.85
		WORKUP	TEMPERATURE	242	0.20	0.16	0.18	0.23	0.19	0.21
			TIME	81	0.00	0.00	0.00	0.00	0.00	0.00
	System				**0.52**	**0.59**	**0.55**	**0.56**	**0.65**	**0.60**
BioBERT-based	ARG1	REACTION_STEP	OTHER_COMPOUND	161	0.08	0.05	0.06	0.08	0.05	0.06
			REACTION_PRODUCT	1,101	0.68	0.56	0.62	0.74	0.63	0.69
			REAGENT_CATALYST	1,272	0.47	0.38	0.42	0.49	0.40	0.44
			SOLVENT	1,134	0.49	0.40	0.44	0.50	0.41	0.45
			STARTING_MATERIAL	1747	0.48	0.73	0.58	0.51	0.80	0.62
		WORKUP	OTHER_COMPOUND	4,097	0.48	0.62	0.54	0.52	0.71	0.60
			REACTION_PRODUCT	11	0.00	0.00	0.00	0.00	0.00	0.00
			SOLVENT	4	0.00	0.00	0.00	0.00	0.00	0.00
			STARTING_MATERIAL	4	0.00	0.00	0.00	0.00	0.00	0.00
	ARGM	REACTION_STEP	TEMPERATURE	813	0.28	0.08	0.12	0.50	0.14	0.22
			TIME	839	0.45	0.44	0.44	0.51	0.50	0.50
			YIELD_OTHER	1,043	0.80	0.87	0.83	0.81	0.89	0.85
			YIELD_PERCENT	937	0.85	0.84	0.84	0.85	0.85	0.85
		WORKUP	TEMPERATURE	242	0.28	0.15	0.20	0.30	0.16	0.21
			TIME	81	0.17	0.02	0.04	0.17	0.02	0.04
	System				**0.52**	**0.54**	**0.53**	**0.56**	**0.60**	**0.58**
ChEMU_2020 teams	Melaxtech Zhang and Zhang (2020)				**0.92**	**0.91**	**0.92**	**0.93**	**0.93**	**0.93**
	NextMove/Minesoft Lowe and Mayfield (2020)				0.85	0.76	0.80	0.87	0.78	0.82
	OntoChem He et al. (2021)				0.80	0.38	0.51	0.84	0.40	0.54
Baseline	ChEMU organizers He et al. (2021)				0.21	0.73	0.33	0.21	0.75	0.33

System performance of three methods (top 3 bold lines). The last bold line shows the best performance out of the chemu teams.

The average performance of the end-to-end system runs slightly lower than the EE system due to the error propagation from our NER. However, we can see the exact match performance of the methods is consistent with the results of our EE-independent evaluation (Table 5). Overall, the CNN-based method obtained a higher exact and relax precision and *F*_1_ score than both the BERT-based methods; however, the BERT_cased method obtained comparatively high recall. We believe the CNN-based method obtained a higher precision than the BERT-based method due to the word embeddings used and the input representation format. ChEMU patent embeddings are trained over patents specifically, and the domain-related information in the patent embeddings provides a better representation of the terms than the contextualized embeddings used in the BERT-based methods. Also, BERT-based methods do not take the positional information of the entities into account, whereas the CNN-based method does. Both BERT-based methods obtain similar precision and *F*_1_ scores, but the BERT_cased method obtains higher recall. The relaxed end-to-end system results show a slight increase in recall and a slight decrease in precision compared to the EE-independent evaluation. Relaxed BiLSTM-CRF scores are comparatively similar to the ground truth (precision—0.95, recall—0.99, and F-score—0.97) for the relaxed evaluation. Hence, the borders of the relaxed NER predictions of the BiLSTM + CRF can include the entity names within the context. We believe these accounts for the slight increase in the recall and slight decrease in precision from the EE evaluation. In addition, both tasks use our BiLSTM + CRF model to identify the trigger words. Since the performance of the trigger word prediction strongly influences the performance of the trigger word–entity pair prediction, we would expect to see a similar performance for both tasks.

Comparing our results, previous works, and baseline shows that both methods obtain a higher precision and *F*_1_ score than the baseline, but not recall. The baseline (He et al., 2021) used a CRF-based model for NER and a rule-based system for EE. All systems outperform the baseline in terms of the *F*_1_ score under both relax and exact matches. Melaxtech (Zhang and Zhang, 2020) outperformed all other systems using a BiLSTM + CRF for NER and a BERT-based method for EE similar to our methods. However, they performed post-processed steps utilizing a set of pattern rules which increased the performance. Thus, we can see that the recall in most of the participants’ systems of the ChEMU_2020 challenge is substantially lower than their precision. However, the recall of our methods is higher than that of our precision.

## 6 Conclusion and Future Work

We explored a BiLSTM + CRF and a BioBERT + CRF method to extract entities and trigger words from the patents. Our results showed that the BiLSTM + CRF method using word embeddings trained over chemical patents obtained the highest results across all entities. We believe utilizing domain-related, non-contextualized patent embeddings improved the performance of utilizing the BERT-contextualized embeddings for word representation, indicating that additional fine-tuning of BERT may be required. The BiLSTM + CRF errors primarily occurred due to the models mislabeling entities annotated as OTHER_COMPOUND for more specific labels, like REACTION_PRODUCT or STARTING_MATERIAL. Additionally, the way that our method predicts entity labels may have contributed to errors with labeling entity spans fully. In the future, we plan to focus on better distinguishing between different types of chemical compounds. We explored rule-based, CNN-based, and two BERT-based methods to extract events from chemical reactions, using our BiLSTM + CRF method with the ChEMU patent embeddings to identify the trigger words. Our results showed that the CNN-based method using word embeddings trained over chemical patents obtained the highest results. In addition, the CNN-based and BERT-based methods obtained comparatively higher precision, especially with the REACTION_STEP classes, as those classes have more instances to train on. Meanwhile, as the rule-based method does not require training, it performed better with WORKUP classes, obtaining a higher recall than the other two methods. In the future, we plan to explore building a hybrid model with both CNN- and rule-based methods to increase performance. Also, we plan to explore graph-based CNNs to facilitate diverse input data representation to improve performance. In addition, we treated the end-to-end system as two independent stages where we perform first NER and then EE. In the future, we plan to explore utilizing a joint learning model to learn both entities better, and trigger words and events simultaneously.

## Data Availability

Publicly available datasets were analyzed in this study. These data can be found here: http://chemu.eng.unimelb.edu.au/.

## Footnotes

1
https://www.ncbi.nlm.nih.gov/pubmed/.

2
https://www.ncbi.nlm.nih.gov/pmc/.

3
https://github.com/NLPatVCU/MedaCy/.

4
https://github.com/NLPatVCU/RelEx/tree/CLEF_2020.

5
https://github.com/keras-team/keras.
